# Risk of congenital anomalies around a municipal solid waste incinerator: a GIS-based case-control study

**DOI:** 10.1186/1476-072X-8-8

**Published:** 2009-02-10

**Authors:** Marco Vinceti, Carlotta Malagoli, Sara Fabbi, Sergio Teggi, Rossella Rodolfi, Livia Garavelli, Gianni Astolfi, Francesca Rivieri

**Affiliations:** 1 CREAGEN – Environmental, Genetic and Nutritional Epidemiology Research Center, Department of Public Health Sciences, University of Modena and Reggio Emilia, Reggio Emilia, Italy; 2 LARMA – Laboratory of Environmental Analysis, Surveying and Environmental Monitoring, Department of Mechanical and Civil Engineering, University of Modena and Reggio Emilia, Modena, Italy; 3 Local Health Unit of Reggio Emilia, Reggio Emilia, Italy; 4 Department of Paediatrics, Santa Maria Nuova Hospital, Reggio Emilia, Italy; 5 IMER Registry, Department of Reproduction and Growth, St. Anna Hospital, Ferrara, Italy

## Abstract

**Background:**

Waste incineration releases into the environment toxic substances having a teratogenic potential, but little epidemiologic evidence is available on this topic. We aimed at examining the relation between exposure to the emissions from a municipal solid waste incinerator and risk of birth defects in a northern Italy community, using Geographical Information System (GIS) data to estimate exposure and a population-based case-control study design. By modelling the incinerator emissions, we defined in the GIS three areas of increasing exposure according to predicted dioxins concentrations. We mapped the 228 births and induced abortions with diagnosis of congenital anomalies observed during the 1998–2006 period, together with a corresponding series of control births matched for year and hospital of birth/abortion as well as maternal age, using maternal address in the first three months of pregnancy to geocode cases and controls.

**Results:**

Among women residing in the areas with medium and high exposure, prevalence of anomalies in the offspring was substantially comparable to that observed in the control population, nor dose-response relations for any of the major categories of birth defects emerged. Furthermore, odds ratio for congenital anomalies did not decrease during a prolonged shut-down period of the plant.

**Conclusion:**

Overall, these findings do not lend support to the hypothesis that the environmental contamination occurring around an incineration plant such as that examined in this study may induce major teratogenic effects.

## Background

The possibility that atmospheric emissions of contaminants by municipal solid waste incinerators leads to adverse effects on the health of exposed populations, and of carcinogenic and teratogenic effects in particular, has been the object of a limited number of studies which yielded conflicting and inconsistent results [1-5]. In fact, incinerators emit a number of pollutants [6], including some suspected or established teratogens such as polychlorinated dibenzo-p-dioxins and -furans (acting as endocrine disruptors [7]) and heavy metals like chromium, cadmium, lead, mercury, nickel and arsenic [8-10]. Moreover, residence near these plants was recently associated with induction of genotoxic effects in humans [11]. These issues are of particular interest since waste incineration is widely used in several developed countries and since birth defects monitoring in the exposed populations has been suggested or adopted as a short-term tool to assess health risks associated to waste management options including incineration [1,2].

In the present study, we studied the extent to which the risk for congenital anomalies varied with maternal exposure to emissions from a modern municipal waste incinerator, by conducting a population-based case-control investigation near a plant with intermittent operation during the study period, and by using a GIS-based approach for exposure assessment and for geographical localization of cases and controls.

## Methods

### Study area

A municipal solid waste incinerator with a capacity of 70.000 tons/year is located in the city of Reggio Emilia, Emilia-Romagna region (extension 232 km^2^, population approximately 150,000). The incinerator consists of two combustion lines that started operating in 1968, and has been equipped since 1992 with a dry scrubbing of flue based on sodium bicarbonate for acidic pollutants gas and since 1994 by an activated carbon device for dioxins, furans and mercury adsorption. This plant stopped its activity in April 20, 2002 due to abnormalities in the combustion process and excess emissions of carbon monoxide and other contaminants, and started to operate once again in June 16, 2005.

**Figure 1:**
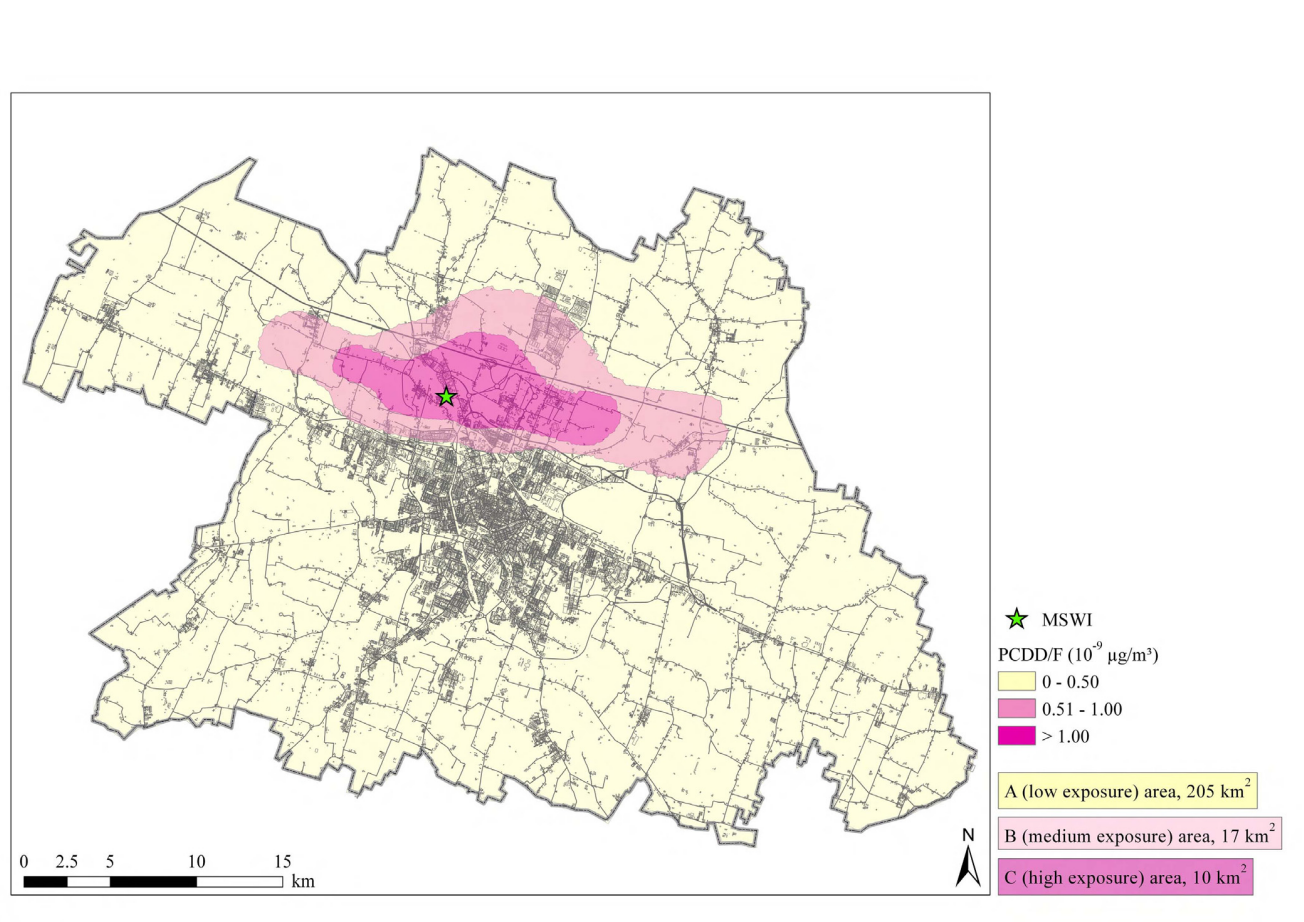
**Map of exposure to polychlorinated dibenzo-p-dioxins and dibenzofurans (PCDD/F) in the city of Reggio Emilia, northern Italy, around the municipal solid waste incinerator (MSWI)**.

We estimated through a dispersion model the average concentrations of dioxin and furans in the lower part of the atmosphere in the city territory, aiming at identifying municipal areas with different amounts of exposure to incinerator emissions at man's height by using the estimated fall-out of polychlorinated dibenzo-p-dioxins and dibenzo-p-furans (henceforth referred to as 'dioxins') as indicators. The dispersion model was computed by using the meteorological database 'CALMET', pre-processor developed by the Emilia-Romagna Region Meteorological Service, for the years 1999, 2000, 2001 (data for 1998 were partially corrupted) and from 1 July 2005 to 30 June 2006. We estimated concentration levels through the model WinDimula 3.0 for Windows [12], an air dispersion model initially developed in the 1980s by Enea (Ente per le Nuove Tecnologie, Energia e Ambiente, Rome) and Maind (Maind s.r.l., Milan) and recently updated [12], based on the Gaussian analytic solution of the turbulent diffusion equation. Its main peculiarity is a special algorithm designed to deal with calm wind conditions that are usual in many Italian regions [13]: the model simulates both short-term and climatological concentrations caused by sources with different geometry, and output concentrations are given on regular grids or on user selected receptors. In the present study, the area over which the computations were made was 20 km × 12 km, with a resolution of 100 m, with the emission source located at (7.022; 6.031) km as respect the South-West corner of the domain. We designed maps of different (A-low, B-intermediate and C-high) ground level exposure to incinerator emissions of dioxins and furans in a Geographic Information System (GIS) environment, using ArcGIS software (version 9.2, ESRI, Redlands, CA 2006) to implement a project georeferenced in the Italian cartographic system (Gauss Boaga) and in the Modena municipality regional technical map layer (Figure 1) and as cutoff points 5 and 10 × 10^-9 ^μg/m^3 ^of dioxins, based on maximum incinerator allowed emission (Figure 1). We also computed a dispersion model predicting heavy metals concentrations, using as cutoff values 0.50 and 1.00 μg/m^3^: the intermediate exposure area was roughly superimposed to C area for dioxins, and these exposure boundaries were not further used in the analysis.

### Study population

We attempted to identify all cases of congenital anomalies in the offspring or in aborted foetuses of women residing in the Reggio Emilia municipality since January 1, 1998 until December 31, 2006. To do this, we used data from the population-based registry of congenital malformations of the Emilia-Romagna Region, named IMER and part of the Eurocat EU program [14], recording since 1979 all cases of abnormalities in live- and stillbirths and since 1996 the induced abortions associated with diagnoses of congenital anomaly observed in the regional hospitals. We also used as additional source of data the Hospital Discharge Directory of Emilia-Romagna residents, available at the Emilia-Romagna Region Health Authority since 1996, and in particular the discharges by Italian hospitals to regional residents reporting an ICD9-CM diagnostic codes from 740.0 to 759.9. These diagnoses were further reviewed by a clinical geneticist (F.R.), and all cases of minor malformations, as defined in accordance to the Eurocat guidelines, were removed from the analysis.

We retrieved control births with their corresponding mothers through random selection within the regional Hospital Discharge Directory. Specifically, to each case we associated a control birth randomly selected among the livebirths without diagnosis of malformations during the same year to women residing in the Reggio Emilia municipality, referred to the same hospital and born in the same year of the matched 'case' mother.

We collected information for both case and referent mothers concerning their residence during the 9 months before parturition (or 3 months for women undergoing induced abortion procedure) and their educational attainment at the Reggio Emilia Municipality General Registry Office. In case of change of residence during the gestational period, we considered for exposure assessment in the present study the woman's residence in the first three months after the estimated date of conception: in the three case showing a change of residence in this early period of the pregnancy, we attributed exposure status on the basis of the longest period of residence within this time span. We then geocoded the retrieved maternal addresses, using the database made available by the Reggio Emilia Province or, in the few cases in which the addresses could not be found in that database, measuring it on site with a geographical positioning system device (Garmin GPSmap 60CSx, Garmin Int. Corp., Olathe, KS). A mother was considered exposed when her address was comprised within the intermediate and high (B and C) exposure areas, after inputting it in the GIS.

All directly and indirectly nominative data were obtained by the Regional Hospital Discharge Registry and by the Reggio Emilia General Registry Office and subsequently analyzed in accordance with the legal and ethical guidelines for personal data protection in epidemiological and scientific research of the Italian law [15] and with the ethical guidelines of the IMER Registry [16].

### Data analysis

**Figure 2:**
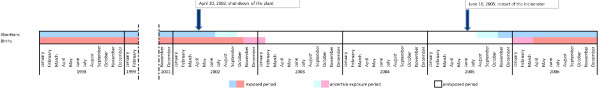
**Operation periods of the municipal waste incinerator of Reggio Emilia, Italy, and corresponding exposure status of cohorts of births and of aborted foetuses undergoing a diagnosis of congenital anomaly during the 1998–2006 period**.

We calculated the prevalence ratio of having a birth or an aborted foetus with a congenital anomaly associated to maternal factors through the odds ratio (OR) with its 95% confidence interval (CI) generated by a conditional logistic regression model, entering as predictive variables the area of residence during gestation, and educational attainment level. Since no point estimate could be generated by the conditional analysis for the anomalies characterized by the lowest prevalence, we used, in these cases, unconditional logistic regression adjusting for both maternal age and education. We carried out this analysis for overall congenital anomalies and for single birth defect categories for the entire study period and, when numbers of cases made it possible, also for the normal operation and shut-down periods of operation of the plant. The former period included all births with congenital anomalies occurring in the periods December 1, 1998–October 31, 2002 and April 1, 2006–December 31, 2006, whilst that ascribed to the shut-down period occurred from February 1, 2003 to December 31, 2005 (Figure 2). The remaining time spans, in the first part of the shut-down period and at the beginning of the reactivation (in 2005) of the plant, were removed from period-specific analysis due to uncertainties in estimating the actual exposure status of women who delivered in these periods. For induced abortions linked to an *in utero *diagnosis of birth defects, the overall time span considered as 'exposed' included the periods January 1, 1998–May 31, 2002 and October 1, 2005–December 31, 2006, whilst abortions considered as 'unexposed' occurred from August 1, 2002 until July 31, 2005, and the events occurred in the remaining periods were removed from analysis. In all these analyses, 'period of exposure' of each control birth was made equal to that of the corresponding matched case. For all statistical analyses we used the package Stata-10 [17].

## Results

Overall, we identified 228 cases of congenital anomalies during the study period: 183 live- and stillbirths presenting one or more defects or congenital syndrome and 45 induced abortions of foetuses with single or multiple anomalies. Maternal age of case mothers (at birth or at time of abortion) ranged from 16 to 44 years; regarding educational attainment level, case and referent mothers who had attended elementary school were 19 (8.33%) and 9 (3.95%) respectively, middle school 83 (36.40%) and 78 (34.21%), high school in 90 (39.47%) and 104 (45.61%) and university 36 (15.79%) and 37 (16.23%).

Using a logistic regression model and adjusting for education and maternal age, OR for congenital anomalies was 1.49 (95% CI 0.70–3.19) in the medium exposure group and 0.66 (95% CI 0.25–1.79) in the high exposure group, compared to the remaining municipal population (Table 1), and results were substantially similar when we used a conditional logistic model (Table 2). As summarized in table 1, grouping together the two highest exposure levels OR in the overall exposed population resulted to be 1.11 (95% CI 0.60–2.04), and when the analysis was carried out for single anomaly categories we found little evidence of excess risk for any disease group and exposure status, with the exception of an increased OR for chromosomal abnormalities in the middle exposure area (OR 2.53, 95% CI 0.88–7.24).

**Table 1:** Prevalence odds ratio for congenital anomalies associated with maternal exposure to the emissions of the incinerator plant of Reggio Emilia, northern Italy, 1998–2006

Area	Cases % (n)	Controls % (n)	Odds ratio^1^
All anomalies			
A (low exposure)	89.0 (203)	90.4 (206)	1.00 (referent)
B (medium exposure)	7.9 (18)	5.3 (12)	1.49 (0.70–3.19)
C (high exposure)	3.1 (7)	4.3 (10)	0.66 (0.25–1.79)
			*P trend 0.881*
Overall exposed area (B+C)	11.0 (25)	9.6 (22)	1.11 (0.60–2,04)

Cardiovascular system			
A (low exposure)	9.6 (87)	90.4 (206)	1.00 (referent)
B (medium exposure)	6.3 (6)	5.3 (12)	0.91 (0.36–2.31)
C (high exposure)	3.1 (3)	4.3 (10)	0.77 (0.22–2.77)
			*P trend 0.666*
Overall exposed area (B+C)	9.4 (9)	9.6 (22)	0.86 (0.40–1.86)

Nervous system			
A (low exposure)	95.7 (22)	90.4 (206)	1.00 (referent)
B (medium exposure)	4.3 (1)	5.3 (12)	0.64 (0.83–4.93)
C (high exposure)	0.0 (0)	4.3 (10)	-^2^
			*P trend 0.344*
Overall exposed area (B+C)	4.3 (1)	9.6 (22)	0.41 (0.05–3.17)

Chromosomal			
A (low exposure)	85.4 (35)	90.4 (206)	1.00 (referent)
B (medium exposure)	12.2 (5)	5.3 (12)	2.53 (0.88–7.24)
C (high exposure)	2.4 (1)	4.3 (10)	0.77 (0.10–6.14)
			*P trend 0.486*
Overall exposed area (B+C)	14.6 (6)	9.6 (22)	1.82 (0.70–4.72)

Genito-urinary			
A (low exposure)	90.4 (19)	90.4 (206)	1.00 (referent)
B (medium exposure)	4.8 (1)	5.3 (12)	0.65 (0.08–5.05)
C (high exposure)	4.8 (1)	4.3 (10)	1.08 (0.14–8.68)
			*P trend 0.904*
Overall exposed area (B+C)	9.6 (2)	9.6 (22)	0.82 (0.18–3.67)

Musculoskeletal			
A (low exposure)	87.2 (34)	90.4 (206)	1.00 (referent)
B (medium exposure)	10.3 (4)	5.3 (12)	1.52 (0.49–4.67)
C (high exposure)	2.5 (1)	4.3 (10)	0.56 (0.07–4.47)
			*P trend 0.928*
Overall exposed area (B+C)	12.8 (5)	9.6 (22)	1.13 (0.41–3.10)

Clefts^2,3^			
A (low exposure)	100.0 (4)	90.4 (206)	1.00 (referent)
Overall exposed area (B+C)	0.0 (0)	9.6 (22)	-

Eye^2^			
A (low exposure)	85.7 (6)	90.4 (206)	1.00 (referent)
Overall exposed area (B+C)	14.3 (1)	9.6 (22)	1.78 (0.20–5.65)

Other and unspecified congenital anomalies^2^			
A (low exposure)	87.5 (14)	90.4 (206)	1.00 (referent)
Overall exposed area (B+C)	12.5 (2)	9.6 (22)	1.11 (0.24–5.10)

^1 ^Estimates computed through unconditional logistic regression (95% confidence interval in parentheses)

^2 ^No point estimate could be computed for the B and C areas

^3 ^No point estimate could be computed for the overall exposed area

**Table 2:** Prevalence odds ratio for congenital anomalies according to maternal exposure to emissions of the municipal solid waste incinerator of Reggio Emilia, northern Italy, 1998–2006 ^1^

	Entire study period			Operation period			Shut-down period		
	Cases % (n)	Controls % (n)	OR (95% CI)	Cases % (n)	Controls % (n)	OR (95% CI)	Cases % (n)	Controls % (n)	OR (95% CI)

All abnormalities									
A area (low exposure)	89.0 (203)	90.4 (206)	1.00 (referent)	91.6 (119)	89.2 (116)	1.00 (referent)	83.5 (71)	90.6 (77)	1.00 (referent)
B area (medium exposure)	7.9 (18)	5.3 (12)	1.55 (0.67–3.56)	6.1 (8)	5.4 (7)	1.10 (0.39–3.06)	11.8 (10)	5.9 (5)	3.17 (0.65–15.46)
C area (high exposure)	3.1 (7)	4.3 (10)	0.67 (0.25–1.77)	2.3 (3)	5.4 (7)	0.41 (0.11–1.61)	4.7 (4)	3.5 (3)	1.30 (0.29–5.82)
			*P trend 0.883*			*P trend 0.321*			*P trend 0.308*
Overall exposed area (B+C)	11.0 (25)	9.6 (22)	1.10 (0.59–2.04)	8.4 (11)	10.8 (14)	0.76 (0.34–1.69)	16.5 (14)	9.4 (8)	2.07 (0.71–6.00)

Cardiovascular anomalies									
A area (low exposure)	90.6 (87)	88.6 (85)	1.00 (referent)	94.9 (56)	83.0 (49)	1.00 (referent)	81.4 (26)	96.9 (31)	1.00 (referent)
B area (medium exposure)	6.3 (6)	6.3 (6)	0.94 (0.27–3.31)	5.1 (3)	8.4 (5)	0.59 (0.14–2.49)	9.3 (3)	3.1 (1)	-^2^
C area (high exposure)	3.1 (3)	5.2 (5)	0.58 (0.14–2.45)	0.0 (0)	8.4 (5)	-^2^	9.3 (3)	0.0 (0)	-^2^
			*P trend 0.494*			*P trend 0.647*			*P trend 0.996*
Overall exposed area (B+C)	9.4(9)	11.5 (11)	0.76 (0.30–1.96)	5.1 (3)	16.8 (10)	0.29 (0.08–1.08)	18.8 (6)	3.1 (1)	-^2^

Nervous system defects									
A area (low exposure)	95.7 (22)	87.0 (20)	1.00 (referent)	100.0 (13)	92.3 (12)	1.00 (referent)	88.9 (8)	77.8 (7)	1.00 (referent)
B area (medium exposure)	4.3 (1)	0.0 (0)	-^2^	0.0 (0)	0.0 (0)	-^2^	11.1 (1)	0.0 (0)	-^2^
C area (high exposure)	0.0 (0)	13.0 (3)	-^2^	0.0 (0)	7.7 (1)	-^2^	0.0 (0)	22.2 (2)	-^2^
			*P trend 0.200*			*P trend 0.995*			*P trend 0.354*
Overall exposed area (B+C)	4.3 (1)	13.0 (3)	0.31 (0.03–3.11)	0.0 (0)	7.7 (1)	-^2^	11.1 (1)	22.2 (2)	0.50 (0.05–5.51)

^1^Odds ratio (OR) with 95% confidence interval (CI) computed with conditional logistic regression

^2 ^No point estimate could be computed

In the period-specific analysis, limited to defects with the highest prevalence, we found little evidence of changes in risk during the shut-down of the plant, since the OR among 'exposed' women was substantially similar to that computed for the operation period (Table 2).

## Discussion

A limited number of epidemiologic studies investigated the risk of congenital anomalies among populations directly exposed to the emissions of waste incinerators [5,18-23]. Some studies did not find an increased prevalence of overall anomalies or of specific groups of birth defects, whilst others detected excess risks for nervous system anomalies [21], cardiovascular defects [21], facial cleft [19,22], urinary defects [22], and overall infant deaths due to congenital malformations [23]. However, methodological limitations considerably hamper the evaluation of results of most investigations: very few studies analyzed maternal residence during the first three months of gestation, or adjusted for maternal age and for socioeconomic status. Moreover, distance of maternal residence (at time of delivery or abortion) from the plant was generally considered in exposure assessment, without taking into consideration the characteristics of the plant (chimney heights and widths, type and amount of combusted waste), the amount of waste combusted and the meteorological factors, with the noticeable exception of the study by Cordier *et al. *[22] who used an exposure index estimated from a Gaussian plume model.

Overall, results of the present study did not suggest the occurrence of an excess teratogenic risk in the vicinity of the incinerator plant, since prevalence increased only in the medium-exposure area and such increase was statistically very unstable, nor any evidence of reduction in risk during shut-down of the plant emerged. Moreover, a more specific analysis for single categories of anomalies did not generally lead us to identify excess risks for any disease group, though these results must be evaluated with caution since most of the computed estimates were statistically unstable. In particular, urogenital anomalies such hypospadias, an abnormality suspected to be associated with parental exposure to environmental endocrine disrupting chemicals [24,25], did not increase in the exposed population. However, we found an excess prevalence of chromosomal anomalies in middle exposure area, which is difficult to interpret since there risk was not increased in the high exposure area, and no association was found in the two epidemiologic studies which specifically examined this category of birth defects [20,22]. We therefore consider it useful to further monitor this finding in the study area or in other comparable contexts.

Some degree of exposure misclassification certainly occurred in the present study. First, since chlorinated compounds are contaminants characterized by persistency in the human body and in the environment, assessment of exposure based on residence during gestation and not on long-term residential history or the occupational environment might have biased to some extent the actual exposure burden experienced by the study subjects during the latest years. However, we checked in a random sample of case and referent women (n = 46, 10% of the study population) their residences three years before date of delivery or abortion, in order to ascertain the extent of long-term changes in exposure status. We found that 36 (78.3%) were then residing in the same exposure area we assigned in the present investigation according to residence at the beginning of pregnancy and 10 (21.7%) immigrated after that date into the municipality (8 towards the low-exposure area and 2 in the high-exposure area), thus suggesting a limited mobility of the study population across the different exposure areas.

Exposure misclassification might have also occurred due to additional sources of dioxins and of heavy metals in the study area, apart from the incineration plant, through different pathways of intake (inhalation, ingestion and dermal contact). However, concerning the first category of contaminants, in the study municipality no major industrial sources of dioxins were located such as electric arc furnaces, cement kilns, and copper and aluminium smelters, whilst the contribution of vehicle fuel combustion and of domestic wood burning to emissions is expected to be substantially lower that waste incineration and more evenly distributed [26,27], thus suggesting that waste incineration was by far the major source of environmental contamination with dioxins in the study area, in line with other observations [28,29]. Concerning exposure to heavy metals, findings from studies examining the specific burden of exposure attributable to waste incineration through measurement of biomarkers of exposure in nearby residents or occupationally-exposed workers are conflicting [30-32], and therefore the risk of exposure misclassification in our study area cannot be entirely ruled out.

Estimating if (and to which extent) exposure to the contaminants emitted by the incinerator actually changed during the study period as a consequence of the interruption in the activity of the incinerator is not easy. Few studies examined the kinetics of dioxins in humans, and half-life of 2,3,7,8-tetrachlorodibenzo-p-dioxin (TCDD) was around 3 years in a Seveso population with a mean age of 24 years [33] and 7–9 years in other selected groups of adults [34]. However, it is unclear if these indications may be extended to dioxins and related compounds other than TCDD, 'low-level' exposures might be much less efficient in the TCDD transfer to the foetus [35], and these kinetics appear also to be markedly influenced by the lipid status of the individuals [34], further complicating this issue. Overall, we estimate that the short-term interruption of waste incineration occurred in our study setting had limited effects on exposure status of the local population, also considering the prolonged half-life of dioxins in soils and in locally grown produces, and therefore we emphasize risk estimates based on the overall follow-up period for dioxin exposure. A separate analysis for each operation period might be more meaningful for heavy metals and for their potential teratogenic effects, since it seems likely that interruption of plant activity markedly decreased exposure to elements such as arsenic, lead and mercury having short half-lives in human blood, in the order of 3–40 days [10,36,37].

Results of the present study must be extended with caution to other contexts and particularly to older incinerators, to the relevant differences in amounts and types of contaminants which may be released into the environment by these plants, owing to the type of wastes combusted and the air pollution control technologies, as well as to potential differences in susceptibility of the exposed individuals. Moreover, we did not examine other reproductive issues apart from congenital anomaly risk such as altered male-to-female birth ratio, low birth weight or twinning, and therefore our investigation cannot be considered a comprehensive assessment of reproductive health in the exposed population.

## Conclusion

In this study setting, maternal exposure to the emissions of a municipal solid waste incinerator, as estimated through a dispersion model, was not associated with excess risk of congenital anomalies in the offspring. These results might not apply to incinerators emitting higher amounts of pollutants such as dioxins and heavy metals.

## Competing interests

One of the authors (MV) has worked as a consultant of the municipal Gas, Waste and Water Agency of Reggio Emilia (currently named "ENIA", formerly "AGAC", mainly owned by the Reggio Emilia Province municipalities) for the for health risk assessment of the city incinerator, of a landfill and of drinking water. However, this Agency has not taken any part in designing, carrying out or funding the present study.

## Authors' contributions

MV and CM designed the study protocol, collected information about cases and controls, carried out data analysis and drafted the manuscript. SF and ST modeled the incinerator emissions and run the GIS database. RR selected potential case mothers through the Emilia-Romagna Region Hospital Discharge Registry and did the matching and sampling of control mothers. LG, FR and GA identified the case mothers eligible for the study from the Emilia-Romagna Region birth defect registry "IMER" and reviewed the cases retrieved through the Hospital Discharge Registry. All authors discussed study results, and read and approved the final manuscript.
